# Optimized conditions for gene transduction into primary immune cells using viral vectors

**DOI:** 10.1038/s41598-023-39597-2

**Published:** 2023-07-31

**Authors:** Yeongrin Kim, Da Yeon Lee, Ji U Choi, Jin Song Park, So Myoung Lee, Chung Hyo Kang, Chi Hoon Park

**Affiliations:** 1 Bio and Drug Discovery Division, Korea Research Institute of Chemical Technology, 141 Gajeong-ro, PO Box 107, Daejeon, 34114 Republic of Korea; 2 College of Pharmacy, Chungnam National University, Daejeon, 34134 Republic of Korea; 3 Medicinal Chemistry and Pharmacology, Korea University of Science and Technology, Daejeon, 34316 Republic of Korea

**Keywords:** Cell biology, Immunology, Molecular biology

## Abstract

Chimeric antigen receptor (CAR) T cell therapy has emerged as a promising modality for anti-cancer treatment. Its efficacy is quite remarkable in hematological tumors. Owing to their excellent clinical results, gene- modified cell therapies, including T cells, natural killer (NK) cells, and macrophages, are being actively studied in both academia and industry. However, the protocol to make CAR immune cells is too complicated, so it is still unclear how to efficiently produce the potent CAR immune cells. To manufacture effective CAR immune cells, we need to be aware of not only how to obtain highly infective viral particles, but also how to transduce CAR genes into immune cells. In this paper, we provide detailed information on spinoculation, which is one of the best known protocols to transduce genes into immune cells, in a methodological view. Our data indicate that gene transduction is significantly dependent on speed and duration of centrifugation, concentration and number of viral particles, the concentration of polybrene, and number of infected immune cells. In addition, we investigated on the optimal polyethylene glycol (PEG) solution to concentrate the viral supernatant and the optimized DNA ratios transfected into 293T cells to produce high titer of viral particles. This study provides useful information for practical production of the gene-modified immune cells using viral vectors.

## Introduction

Due to its excellent efficacy in clinical trials, chimeric antigen receptor (CAR) T cell therapy is firmly positioned as an innovative modality for anti-cancer treatment^1^. Recently, genetically engineered natural killer (NK) cells and macrophages with CAR have shown promising results in clinical and preclinical studies^2,3^. However, complicated and difficult processes for the preparation of CAR immune cells make it difficult for this therapy to be applied to patients in need. The production of CAR T cells involves a variety of steps. The patient’s white blood cell (WBC) should be isolated through leukapheresis. Primary T cells were then expanded and activated for approximately 1–2 weeks. During this step, CAR gene is transduced into T cells. Once quality control of CAR T cells is achieved, patients are injected with CAR T cells following lymphodepleting chemotherapy.

Most manufactured CAR T cells used in clinical trials are made by retroviral or lentiviral vectors^4^. These vectors are successfully integrated into the genome resulting in long term expression of CAR. Lentivirus can infect both non-dividing and dividing cells, whereas retrovirus can infect only dividing cells. Although non-viral methods for gene transduction into immune cells are under investigation, most of the CAR T cells in current clinical trials are still made by viral particles^5^. In CAR T cells made by viral vectors, the anti-tumor activities depend on how infective the viral particles are. On top of that, the method to introduce the viral vectors into T or NK cells should be optimized to produce potent CAR T cells. Therefore, many studies on lentiviral or retroviral particles as a means to deliver CAR genes into cells have been published^6–14^. However, it is still unclear how to prepare high quality viral vectors and efficiently transduce these genes into immune cells.

Here, we studied on the way to efficiently prepare the gene modified immune cells efficiently using lenti or retro viral vectors. We provided information for making infective viral particles and the way to transduce the genes into immune cells to maximize gene expression.

## Results

### The optimal centrifugation conditions in spinoculation for gene transduction into immune cells

**Figure 1:**
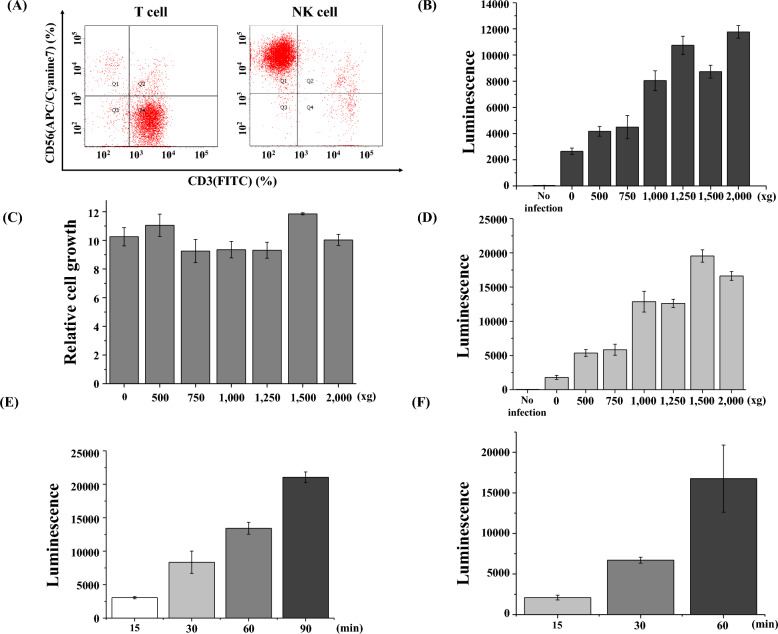
(**A**) The expanded T- or NK cells were analyzed using FACS analysis with CD3 and CD56 antibodies. (**B**,**C**) PBMCs were cultured with CD3/CD28 dynabeads (human T-cell activator) for 7 days (7d), then the VSVg-pseudotyped lentiviral luciferase gene was transduced into T-cells at different g forces. After 4 days (11d), luminescence and cell numbers were measured. (**D**) Primary NK cells were activated by co-culture of PBMCs with aAPCs. After 3 days, the RDF-pseudotyped retroviral luciferase gene was transduced into NK cells through spinoculation at different g forces. Luminescence was measured 4 days after gene transduction. (**E**) After T-cell activation, RDF-pseudotyped retroviral luciferase gene was transduced into T-cells through spinoculation at different centrifugation times. Luminescence was measured 24 h after gene transduction. (**F**) Primary NK cells were activated by co-culture of PBMCs with aAPCs. After 3 days, the RDF-pseudotyped retroviral luciferase gene was transduced into NK cells through spinoculation at different times. Luminescence was measured 7 days after gene transduction.

In this study, we expanded T cells and NK cells using CD3/CD28 dynabeads and feeder cells, respectively (Fig. 1A). There are several methods to transduce genes into immune cells including T cells, NK cells, and macrophages. One of the most popular ways is spinoculation. We investigated the optimal conditions for spinoculation. For gene transduction into T cells, peripheral blood mononuclear cells (PBMCs) were activated with CD3/CD28 dynabeads, then centrifuged at different ‘*g*’ forces with vesicular stomatitis virus G (VSV-G) pseudotyped luciferase lentiviruses. The luminescence values indicate how effectively the genes were transduced into the cells. As *g* force increased, the transduction efficiency was improved as well (Fig. 1B). Spinoculation with 2000×*g* enhanced transduction efficiency by approximately sixfold compared to transduction without spinoculation. It was concerned that a high g force might damage T cells to impair the cell growth. However, Fig. 1C shows that cell growth was not reduced even when a 2000×*g* force is applied. We have performed the same experiment with NK cells. To prepare primary NK cells, PBMCs were co-cultured with artificial antigen presenting cells (aAPCs), K562 cells expressing both membrane bound IL21(mbIL21) and 41BBL. NK cells were centrifuged with RDF pseudotyped luciferase retroviruses (Fig. 1D). Similar to T cells, gene transduction into NK cells increased as the *g* force increased, and the proliferation of NK cells was not damaged by a high g force (data not shown). We also examined the impact of the duration of spinoculations on gene transduction into T and NK cells (Fig. 1E,F). In T cells, spinoculation for 90 min showed best transduction. Also, in NK cells, spinoculation for 60 min, which is the longest time in this experiment, made best transduction. Figure 1E,F show that the gene transduction efficiency is proportional to the spinning time.

### Characteristics of the viral particles

**Figure 2:**
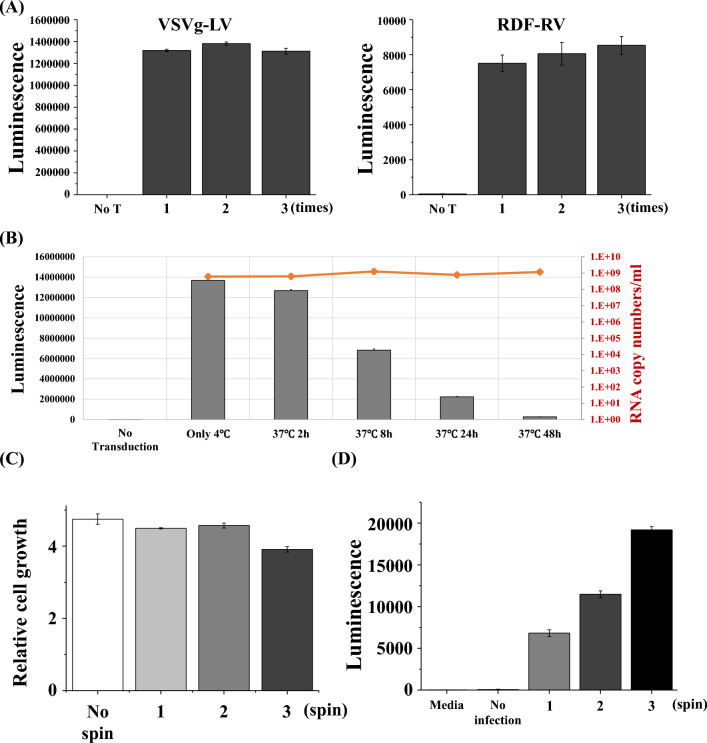
(**A**) VSVg-pseudotyped lentivirus or RDF-pseudotyped retrovirus went through one, two, or three rounds of the freeze-and-thaw cycle. 293T- or T-cells were transduced with lentiviral or retroviral luciferase gene in the presence of polybrene, respectively. After 2 days, luminescence was measured. (**B**) VSVg-pseudotyped lentiviral particles remained for 2, 8, 24, or 48 h in 37 at °C, then used for luciferase gene transduction into 293 T-cells. After 3 days, luminescence was measured. Viral RNA copy numbers were measured using the qRT-PCR kit. Blue bars or an orange line indicates luminescence or viral RNA copy numbers, respectively. (**C**) Primary T-cells went through one, two, or three rounds of centrifugation at 1200*g* for 90 min, then cell population was measured using the CellTiter-Glo assay. (**D**) Primary T-cells were infected with luciferase lentivirus through one, two, or three rounds of centrifugation at 1200*g* for 90 min, then luminescence was measured.

Viral particles are regarded to be easily damaged by multiple freeze-and-thaw. To verify this, we tested the infectious ability of the VSV-G pseudotyped lentivirus and RDF pseudotyped retrovirus, which underwent freeze-and-thaw once, twice, or three times. As shown in Fig. 2A, the infectious abilities were the same regardless of the number of freeze-and-thaw cycles. This means that freeze-and-thaw doesn’t weaken the infectivity of lentiviruses and retroviruses. Temperature is also regarded as an integral factor in determining the viral infectious ability. We stored the VSV-G pseudotyped lentiviral particles at 4 °C overnight, 37 °C for 2 h, 8 h, 24 h, or 48 h before gene transduction into 293 T cells. Our data showed that the longer the viral particles remained at 37 °C, the less infectious the viral particles were (Fig. 2B). Surprisingly, the viral RNA copy numbers, which were determined by titration kit of qRT-PCR, were the same among all samples. This means that the qRT-PCR titration kit cannot differentiate between infective and the non-infective viral particles.

To make T cells express more CAR protein, some laboratories perform spinoculations several times. We tested whether more than one round of centrifugation impaired T cell growth. As shown in Fig. 2C, multiple centrifugation did not negatively affect T cell growth. As we expected, multiple centrifugation enhanced the gene transduction into T cells (Fig. 2D).

### The condition for gene transduction using viral particles

**Figure 3:**
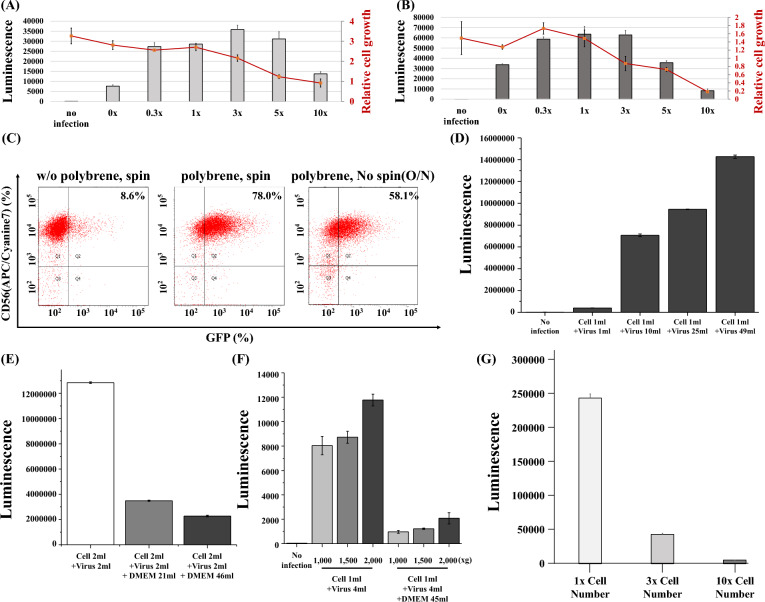
(**A**,**B**) T- or NK cells were activated by co-culture of PBMCs with CD3/CD28 dynabeads or aAPCs, respectively. After 3 days, RDF pseudotyped retroviral luciferase genes were transduced into T-cells (**A**) or NK cells (**B**) under different polybrene concentrations. Luminescence and cell numbers were measured 48 h after gene transduction. The 1 × represents 40 µg/ml of polybrene. Gray bars or orange graph indicates luminescence or relative cell number, respectively. (**C**) NK cells were transduced with RDF-pseudotyped retroviral GFP genes with or without polybrene and spinoculation. After 1 week, FACS analysis was performed. (**D**) 293T-cells were infected with different volumes of VSVg-pseudotyped luciferase lentiviruses. After 4 days, luminescence was measured. (**E**) 293 T-cells were infected with different concentration of VSVg-pseudotyped luciferase lentiviral particles. After 5 days, luminescence was measured. (**F**) PBMCs were cultured with CD3/CD28 dynabeads to proliferate T-cells. After 7 days (7d), VSVg-pseudotyped luciferase lentiviral particles were infected into activated T-cells at different concentrations of viral particles and at different g forces. After 4 days (11d), luminescence was measured. (**G**) Different numbers of T-cells were infected with VSVg-pseudotyped luciferase lentiviruses, whose RNA copy number was 1.7 × 10^10^. After 3 days, luminescence was measured.

Polybrene, which neutralizes charge repulsion between the cell surface and virions, is widely used for gene transduction with viral particles. We checked whether high concentrations (3×, 5×, 10×) of polybrene could enhance gene transduction into immune cells. Figure 3A shows that excessive amounts of polybrene did not improve gene transduction in T cells. Rather, a high concentration of polybrene reduced T cell viability. The same was true for NK cells. High concentrations of polybrene deteriorated NK cell proliferation without improving gene transduction (Fig. 3B). Fluorescence-activated cell sorting (FACS) data shows the importance of polybrene in gene transduction into NK cells (Fig. 3C). In spinoculation, polybrene significantly enhanced the transduction efficiency. In the presence of polybrene, gene is transduced into NK cells by co-culture of NK cells with retroviral vectors for 24 h. However, incubation of NK cells with polybrene for 24 h damaged the NK cell viability (data not shown). Next, we checked whether transduction efficiency was dependent on either the number of viral particles or concentration of viral particles. Figure 3D shows that the higher the number of viral particles, the higher the transfection efficiency, which likely indicates the importance of the viral particle numbers. To confirm this, we infected 293T or T cells with the same number of viral particles in different volumes of the cell and virus mixtures. Under these conditions, each mixture had a different concentration of viral particles, although they had the same number of viral particles. As shown in Fig. 3E (293T) and 3F (T cell), high concentrations of viral particles led to superior gene transduction. Therefore, we concluded that both the number and concentration of viral particles are important for gene transduction. We also tested whether gene transduction into T cells was enhanced by decreasing the number of T cells. As shown in Fig. 3G, gene transduction decreased significantly when the number of target cells increased. The number of target cells is also important for efficient gene transduction using viral particles.

### Duration of transduced gene expression in immune cells

**Figure 4:**
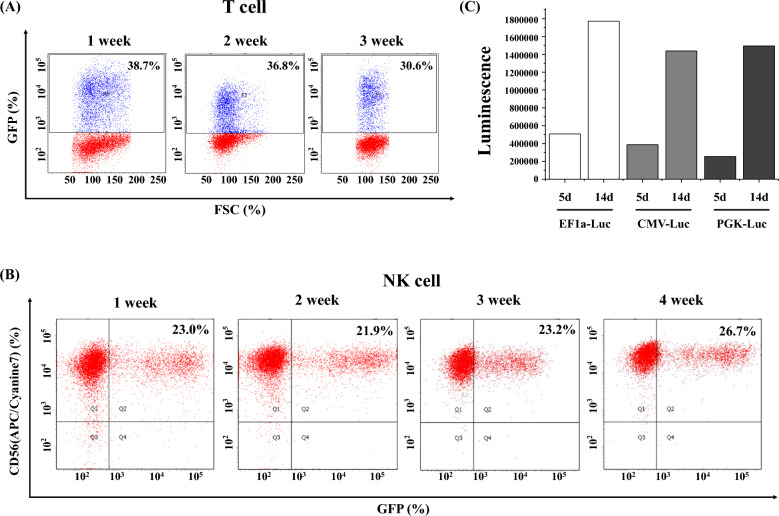
(**A**) PBMCs were activated with CD3/CD28 dynabeads for T-cell activation, then transduced with EF1α promoter driven GFP gene using VSVg-pseudotyped lentiviral particles. FACS analysis was performed every week for 3 weeks. (**B**) NK cells were activated by co-culture of PBMCs with aAPCs. After 2 days, MSCV promoter driven GFP gene was transduced by RDF-pseudotyped retrovirus. FACS analysis was performed every week for 1 month. (**C**) Macrophages were isolated from human PBMCs using a monocyte isolation kit. After 7 days, BaEV-pseudotyped lentiviral luciferase gene was transduced into macrophages. Luminescence was measured after 5 days (5d) or 14 days (14d).

We investigated the duration of transduced gene expression in each immune cell. The elongation factor-1 (EF1) promoter-driven green fluorescence protein (GFP) gene was transduced into T cells using VSV-G pseudotyped lentiviral particles. We performed FACS analysis with T cells every week. We found that the transduced gene was expressed consistently for at least 3 weeks in primary T cells (Fig. 4A). We performed the same experiment using NK cells. For gene transduction into NK cells, we used RDF pseudotyped retroviral particles of murine stem cell virus (MSCV) promoter driven GFP. We found that the transduced gene was also expressed consistently for at least 1 month in primary NK cells. For gene expression in macrophages, we tested several promoters, including the EF1α, cytomegalovirus (CMV), or phosphoglycerate kinase 1 (PGK) promoter. The expression levels of each promoter-induced luciferase gene were similar (Fig. 4C). Moreover, in contrast to T or NK cells, the expression of transduced genes in macrophages increased with time.

### The choice of envelope protein and virus type for gene transduction into immune cells

Lentiviral and retroviral particles are most commonly used for gene transduction into immune cells. To maximize gene transduction by the viral system, it is critical to choose an appropriate envelope protein for each immune cell. In this study, we identified the envelope proteins that were most suitable for gene transduction into each immune cell. In this study, we tested VSVg and RDF.

**Figure 5:**
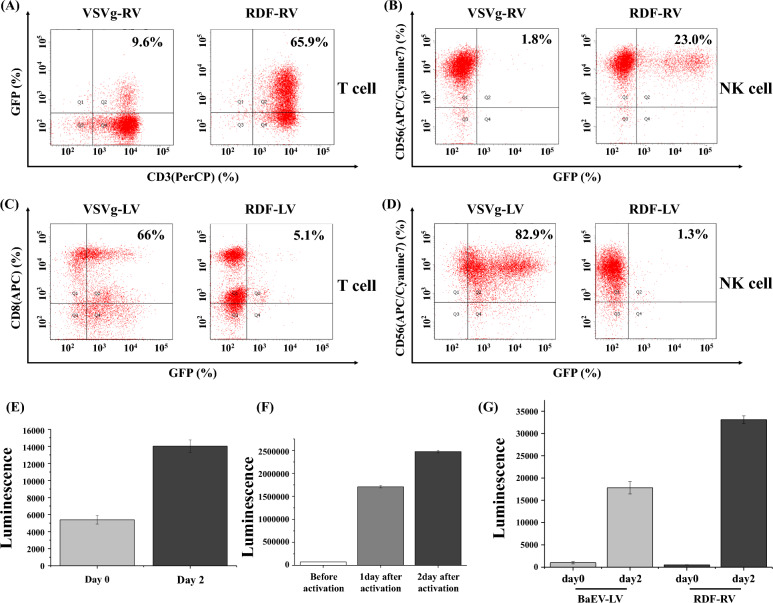
(**A**,**C**) T-cells were activated by co-culture of PBMCs with CD3/CD28 dynabeads. After 48 h, the GFP gene was transduced by VSVg-, or RDF-pseudotyped lentiviruses or retroviruses into T-cells. FACS analysis was performed 7 days after gene transduction. (**B**,**D**) NK cells were activated by co-culture of PBMCs with aAPCs. After 48 h, GFP gene was transduced by VSVg-, or RDF-pseudotyped lentiviruses or retroviruses into NK cells. FACS analysis was performed 7 days after gene transduction. (**E**) VSVg-pseudotyped luciferase lentiviral particles were infected into T-cells before or 2 days after T-cell activation. Luminescence was measured 5 days after the last gene transduction. (**F**) RDF-pseudotyped luciferase retroviral particles were infected into T-cells before or one day to two day after T-cell activation. Luminescence was measured 2 days after the last gene transduction. (**G**) BaEV-pseudotyped luciferase lentiviral particles or RDF-pseudotyped luciferase retroviral particles were infected into NK cells before or 2 days after NK cell activation. Luminescence was measured 5 days after the last gene transduction.

RDF is more suitable than VSVg for the preparation of retroviral vectors for gene transduction into T-cells. As shown in Fig. 5A,B, GFP expression by the RDF pseudotyped retrovirus was much higher than that by the VSVg pseudotyped retrovirus. Contrary to retrovirus, GFP expression by VSVg pseudotyped lentivirus was much higher than RDF pseudotyped lentivirus in both T and NK cells (Fig. 5C,D).

Next, we were curious about when transduction should be performed into immune cells. The VSV-G pseudotyped lentiviral luciferase gene was transduced into T cells before or 2 days after T cell activation using dynabeads (Fig. 5E). Luminescence data indicated that gene transduction after T cell activation increased gene expression by approximately twofold. However, if you transduce the retroviral gene before T cell activation, you will get almost nothing (Fig. 5F). Therefore, retroviral genes should be transduced after T cell activation. We performed the same experiment using NK cells. Figure 5G strongly suggests that gene transduction into NK cells should be performed after cell activation, regardless of whether lentiviruses or retroviruses are used.

### The method for virus concentration with PEG

**Figure 6:**
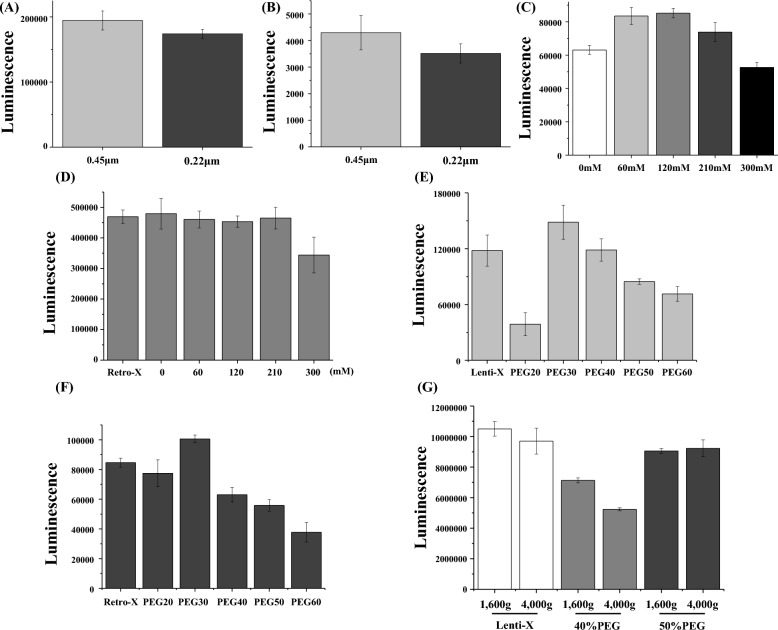
(**A**,**B**) VSVg-pseudotyped luciferase lentiviral particles or RDF-pseudotyped luciferase retroviral particles were filtered. The luciferase gene was transduced into 293T-cells, then luminescence was measured after 2 days. (**C**) VSVg-pseudotyped luciferase lentiviral particles were concentrated using 50% PEG under different NaCl concentrations. Concentrated VSVg-pseudotyped luciferase lentiviral particles were infected into 293T-cells. Luminescence was measured after 2 days. (**D**) RDF-pseudotyped luciferase retroviral particles were concentrated using 30% PEG under different NaCl concentrations. Concentrated RDF-pseudotyped luciferase retroviral particles were infected into 293T-cells. Luminescence was measured after 24 h. Retro-X represents the commercially available retrovirus concentrator. (**E**) VSVg-pseudotyped luciferase lentiviral particles were concentrated using different PEG percentages and 120 mM NaCl. Concentrated VSVg-pseudotyped luciferase lentiviral particles were infected into 293T-cells. Luminescence was measured after 2 days. Lenti-X represents the commercially available lentivirus concentrator. (**F**) RDF-pseudotyped luciferase retroviral particles were concentrated using different PEG percentages without NaCl. Concentrated RDF-pseudotyped luciferase retroviral particles were infected into 293T-cells. Luminescence was measured after 2 days. (**G**) Different g forces, 1600*g* and 4000*g*, were applied to concentrate the VSVg-pseudotyped lentiviral particles. Concentrated VSVg-pseudotyped luciferase lentiviral particles were infected into 293T-cells. Luminescence was measured after 2 days.

Polyethylene glycol (PEG) is widely used to concentrate viral soup. In this method, the virus solution mixed with PEG and NaCl was centrifuged at 1600×*g*. Here, we investigated the best PEG percentage and NaCl concentration for virus concentration. Before virus concentration, the viral soup was filtered to remove 293 T-cell debris. After filtration of the viral soup, we measured the infectious ability of lentiviral or retroviral particles against 293T-cells. The diameter of the lentivirus or retrovirus is approximately 100 nm. As shown in Fig. 6A,B, lentiviral and retroviral particles filtered using a 0.45 µm pore size exhibited higher infectious ability than those filtered using a 0.22 µm pore size. This data indicates that a smaller amount of viral particles pass through the 0.22 µm filter. Next, we tested the optimal NaCl concentration for concentrating the viral particles. With regard to VSV-G pseudotyped lentiviral particles, 60–120 mM NaCl was the best virus concentration (Fig. 6C). NaCl is not required to concentrate RDF pseudotyped retroviral particles (Fig. 6D). We also determined the best PEG percentage for virus concentration. One volume of PEG solution was added to three volumes of viral soup. A 30% PEG solution (PEG30) was the best for concentrating VSVg pseudotyped lentiviral particles or RDF pseudotyped retroviral particles (Fig. 6E,F). We checked whether the increased g force enhanced the concentration of viral particles. We centrifuged the PEG-viral particle solution at 1600*g* or 4000*g*. Interestingly, the 4000*g* force did not improve the virus concentration compared to the 1600*g* force (Fig. 6G).

### Optimal DNA amounts for production of lentiviral particles

**Figure 7:**
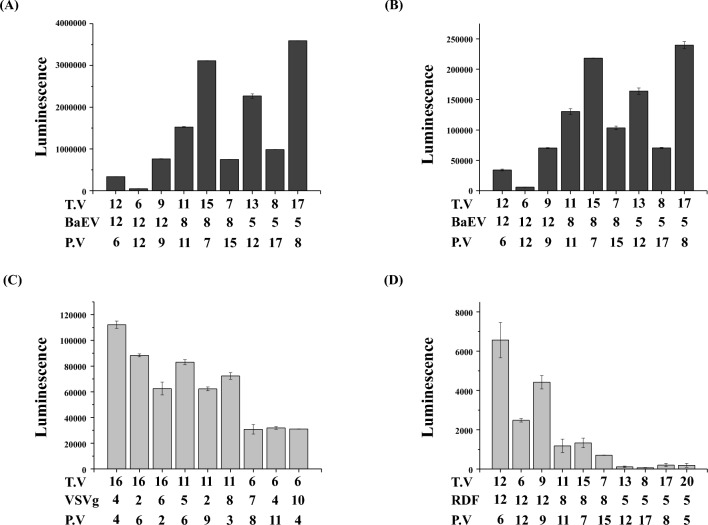
(**A**,**B**) To produce BaEV-pseudotyped lentiviral particles, different amounts of transfer vector (T.V), BaEV, and packaging vector (P.V) were co-transfected into 293T-cells. Primary T- (**A**) or NK (**B**) cells were infected with these lentiviral particles, then luminescence was measured after 4 days. (**C**) To produce VSVg-pseudotyped lentiviral particles, different amounts of transfer, VSVg, and packaging vector were co-transfected into 293T-cells. Primary T-cells were infected with these lentiviral particles, then luminescence was measured after 4 days. (**D**) To produce RDF-pseudotyped retroviral particles, different amounts of transfer, RDF, and packaging vector were co-transfected with 293T-cells. Primary T-cells were infected with these retroviral particles, then luminescence was read after 4 days.

The mass ratio of lentiviral or retroviral DNAs is known to be critical to produce infective viral particles. Different amounts of lentiviral or retroviral DNA, including transfer, envelope, and packaging vectors were transfected into 293T cells to produce pseudotyped viral particles. T- or NK cells were infected with each luciferase viral particle and luminescence was measured. As shown in Fig. 7A,B, 17:5:8 or 15:8:7 ratio was best for producing infective baboon endogenous virus (BaEV) pseudotyped lentiviral particles. A 6:12:12 ratio rarely produces infective viral particles. For VSVg pseudotyped lentiviral particles, 16:4:4 was the best (Fig. 7C). We also investigated for producing retroviral particles. As shown in Fig. 7D, the 12:12:6 ratio was best for producing RDF pseudotyped retroviral particles, whereas this ratio showed poor production of BaEV pseudotyped lentiviruses. Our data demonstrates that the productions of pseudotyped viral particles is highly dependent on the viral DNA ratio.

## Discussion

Manufacturing CAR T-cells involves complicated processes; therefore, many studies have been conducted to prepare high-quality CAR T-cells^15–18^. Among the many steps in manufacturing CAR T-cells, the preparation of high-quality lentiviral or retroviral particles and setting up the optimized conditions to transduce CAR genes into immune cells are critical steps. To obtain viral particles, viral plasmids, including the transfer, packaging, and envelope vector, should be transfected into 293T-cells. Our data shows that the mass ratio of each viral plasmid transiently transfected into 293T-cells is critical for infectious viral particle production (Fig. 7). Interestingly, infective pseudotyped viral particle production varied significantly according to the mass ratio of viral plasmids. Each pseudotyped viral particle had its own preferred DNA ratio. Therefore, we recommend that each lab should perform DNA ratio optimization before making viral particles from 293T-cells. It is also important to concentrate the viral particles from a large volume of the viral supernatant to transduce CAR genes into immune cells. There are several methods to achieve this goal. Here, we optimized the conditions for viral particle concentration in a PEG solution (Fig. 6). Although most public protocols for viral particle concentration focus only on the PEG percentage, our data demonstrate that the concentration efficiency is dependent on NaCl concentration as well. A 120 mM NaCl concentration maximized the concentration of VSVg-pseudotyped lentivirus (Fig. 6C). However, no NaCl was required to concentrate the RDF-pseudotyped retrovirus (Fig. 6D). Therefore, it is necessary to use different solutions to concentrate different viral particles. Interestingly, a high g force (4000×*g*) did not guarantee a high concentration of viral particles in the PEG method (Fig. 6G).

Spinoculation is a popular method for transducing genes into immune cells. In many CAR T cell papers for clinical trials, they use spinoculation for gene transduction^19,20^. Several papers proved that spinoculation enhanced the gene transduction into T cells^21–23^. Spinoculation was reported to trigger dynamic actin and cofilin activity to facilitate HIV-1 infection into T cells^24^. There are several agents to assist the gene transduction into human primary cells including polybrene, vectofusin, and retronectin^23,25^. Our data shows optimal concentration of polybrene, one of these agents, is inevitable to increase the gene transduction in spinoculation. In T- and NK cells, the transduction efficiency increased as the g force increased (Fig. 1). Fortunately, a high g force does not suppress T- or NK cell proliferation. In addition, Fig. 1F indicates that the longer the spinoculation, the better the gene transduction both in T and NK cells. Our study demonstrated that both the absolute number and the number per volume of viral particles are important in spinoculations (Fig. 3D–F). Gene transduction was enhanced as the number of viral particles increased. In addition, the smaller the volume of the mixture of cells and viral particles is, the higher the transduction efficiency. For example, 100 viral particles in 1 mL were more infectious than 100 viral particles in 10 mL. Therefore, we not only need to increase the number of viral particles but also reduce the volume of the viral particle solution to maximize the transduction efficiency.

Viral particles have been considered to be vulnerable to the freeze-and-thaw cycles. In this study, we investigated the extent to which freeze-and-thaw impaired the infectivity of lentiviral particles (Fig. 2). Surprisingly, several rounds of freeze-and-thaw didn’t impair the viral particles. This was an unexpected result. Viral particles are also known to be vulnerable to high temperature. In our study, viral particles stored at 37 °C significantly lost their infectivity compared to viral particles stored at 4 °C (Fig. 2B). Therefore, viral particles must be kept at a low temperature to maintain their infectivity.

Filtration of viral supernatant is essential to get rid of viable 293T cells. It is known that the diameter of viral particle is 80–100 nm. Therefore, we need to be careful to select the pore size of filter for viral supernatant filtration. Previous study reported that the recovery of viral particles after filtration is dependent on the filter pore size^26^. Our data also demonstrated that the filter with 0.22 µm pore size reduced the recovery of viral particles compared to filter with 0.45 µm pore size.

We examined the duration of expression of the transduced genes (Fig. 4). The expression levels of the transduced gene in T-cells or NK cells were consistent for at least 1 month. Next, we determined which pseudotyped viral particles were appropriate for gene transduction into T-cells or NK cells (Fig. 5). Interestingly, VSVg-pseudotyped retroviruses were observed as not effective for gene transduction into T-or NK cells. In contrast to retroviruses, lentiviral particles prefer the VSVg for gene transduction. To obtain the best expression of the transduced gene in T- or NK cells, we must also consider when transduction by viral particles is to be conducted. As shown in Fig. 5F–H, regardless of the virus used, transduction should be done 1–2 days after T- or NK cell activation.

Lin et al. investigated the optimal condition for gene transduction into mesenchymal stem cells (MSCs)^27^. They reported that spinoculation increased the gene transduction into MSCs, and virus concentration is critical factor for gene transduction into MSCs. Interestingly, in MSCs, the cell number transduced with lentivirus is not important for the gene transduction efficiency, which is inconsistent with our results in T cells (Fig. 3G). Optimization for gamma-retroviral gene transfer in T cells using spinoculation was investigated^28^. In this study, they demonstrated that spinoculation without retronectin significantly reduced gene transduction. Our data shows the importance of polybrene in gene transduction by spinoculation. However, we need to be careful to use polybrene, because incubation of T or NK cells with polybrene overnight severely diminished the cell viability (data not shown). One of the advantages of spinoculation is that the incubation of immune cells with polybrene can be minimized to less than 2 h.

In this study, we aimed to enhance gene transduction into immune cells. CAR proteins should be abundantly expressed by efficient gene transduction to make CAR T- or NK cells potent against tumors. We hope that this paper will help those who manufacture effective CAR T- or NK cells for academic or clinical applications.

## Materials and methods

### PBMC isolation

Human peripheral blood mononuclear cells (PBMCs) were obtained from healthy donors who provided written informed consent according to protocols approved by Korea National Institute for Bioethics Policy Institutional Review Board (approval no. P01-201607-31-003). All experimental protocols using PBMCs were approved by IRB. All methods using blood samples were performed in accordance with the institutional biosafety guidelines. PBMCs were diluted using an equal volume of PBS containing 2% FBS (Hyclone, 16000-044) solution. Once blood was diluted the, 30 ml of diluted blood was gently paced over 15 ml of lymphoprep density gradient medium (Stemcell, 07851/07861) in a 50 ml conical tube. The layered solution was centrifuged at 800×*g* for 20 min at room temperature (15–25 °C). After centrifugation, the upper plasma layer was discarded without dispersing the red blood cells. A layer of granulocyte pellets and mononuclear cells, which were located between the upper plasma layer and the second layer of the lymphoprep medium were collected using a pipette. The collected PBMCs were washed twice using PBS containing 2% FBS at 300*g* for 8 min at room temperature. PBMCs were counted and resuspended in a freezing medium composed of 90% FBS and 10% DMSO. PBMCs were cryopreserved in liquid-nitrogen tanks (1–3 × 10^7^ cells/vial).

### T cell activation and culture

Cryopreserved PBMCs were quickly thawed in a 37℃ water bath. PBMCs were cultured in RPMI-1640 medium supplemented with 10% FBS and 200 IU/ml human recombinant IL-2 (R&D Systems, 202-IL) and activated using human T-activator CD3/CD28 dynabeads (Gibco, 11132D) at a ratio of 1:1 (beads:T-cells). T-cells were cultured for 7–14 days by adding fresh medium and human IL-2 every 3 days.

### NK cell activation and culture

Cryopreserved PBMCs were quickly thawed in a 37 °C water bath. PBMCs were cultured in RPMI-1640 medium supplemented with 10% FBS and 200 IU/ml human recombinant IL-2 (R&D Systems, 202-IL) and activated using irradiated K562 (mbIL21-41BBL) feeder cells at a ratio of 1:1 (feeder cells:NK cells). NK cells were cultured for 7–14 days while adding fresh medium and human IL-2 every 3 days.

### Macrophage isolation, and macrophage culture

The EasySep Human Monocyte Isolation Kit (Stemcell, 19359) was used. Cryopreserved PBMCs were quickly thawed in a 37 °C water bath. PBMCs were resuspended at a density of 5 × 10^7^ cells/ml in 1 ml of RPMI-1640 culture medium containing 10% FBS. The cells were transferred into a 5 ml polystyrene round-bottom tube and then a 50 µl of the isolate cocktail was added. The mixture was incubated for 5 min at room temperature. A volume of 50 µl of magnetic particles were added to the mixture and incubated for 5 min at room temperature. After adding 1.5 ml culture medium into the sample tube, the mixture tube was placed in a magnet and incubated at room temperature for 3 min. The cell suspension was transferred into a new tube, and 7.5 ml of RPMI-1640 medium supplemented with 50 ng/ml GM-CSF (Peprotech, 300-03) was added.

### Spinoculation

For gene transduction into T- or NK cells by spinoculation, the cells were centrifuged with lentiviral or retroviral supernatant containing 8 µg/ml polybrene for 90 min at 1000–2000*g* at room temperature. After centrifugation, the viral medium was removed and the cells were cultured in RPMI-1640 medium supplemented with 10% FBS and 200 IU/ml IL-2. For macrophage spinoculations, macrophages were detached from culture dishes using a cell scraper, transferred into a lentivirus solution containing 8 µg/ml polybrene, and centrifuged for 90 min at 1000*g* at room temperature. After centrifugation, the viral soup was removed and the cells were cultured in RPMI-1640 medium supplemented with 10% FBS and 50 ng/ml GM-CSF.

### Luminescence

A volume of 100 µl of luciferase expressing cells were transferred into 96-well plates. Cells were mixed with an equal volume of bright-glo assay solution (Promega, E2620) and incubated at room temperature for 10 min with shaking. Half of the mixture was transferred into a 96-well white opaque plate. The luminescence signal was read using an EnSpire alpha (PerkinElmer) detector.

### Production and titration of pseudotyped lentiviruses or retroviruses from 293T-cells

For lentivirus generation, psPAX packaging vector, and pMD2.G -VSVg, pRDF, BaEV envelope vectors, and pLVX-luciferase transfer vectors were used. For retrovirus production, the pEQ-PAM-3E packaging vector, pMD2.G -VSVg, pRDF, or BaEV envelope vectors, and MSCV-luciferase transfer vectors were used. A total of 7 × 10^6^ of 293T-cells were seeded in 100 mm culture dishes. 293T-cells were transfected with 15 µg, 8 µg, and 7 µg of the transfer, envelop, and packaging plasmid, respectively, for lentivirus production using calcium phosphate. For retrovirus production, 293T-cells were transfected with 12 µg, 12 µg, and 6 µg of the transfer, envelop, and packaging plasmid. Viral soup was collected two times every 24 h, then filtered through a 0.45 µm PES filter (Millipore, SLHPR33RS). To determine the titer of viral particles, Lenti-X qRT-PCR Titration Kit (Clontech, 631235) was used.

### Virus concentration

The viral supernatant was filtered through a 0.45 µm PES filter. Ten ml of 30% PEG and 1 ml of 4 M NaCl were added to 30 ml viral solution. The mixture were mixed gently by pipetting and incubated at 4 °C for at least 5 h. The mixture was centrifuged at 1800*g* for 60 min at room temperature. After centrifugation, the supernatant was removed without disturbing the pellet (visible off-white pellets). The white pellet was resuspended in 1 ml of DMEM medium, then used immediately or stored at − 80 °C.

## Data Availability

Data supporting this study are available to any researcher. Please contact by e-mail (chpark@krict.re.kr).
